# Experiences of and Interventions for Adult Survivors of Childhood Sexual abuse in South Asia: A systematic review

**DOI:** 10.1192/j.eurpsy.2023.2085

**Published:** 2023-07-19

**Authors:** S. Talwar, C. Osorio, R. Appleton, J. Billings

**Affiliations:** 1 Psychiatry; 2 University College London, London, United Kingdom

## Abstract

**Introduction:**

Adult survivors of childhood sexual abuse (CSA) may experience psychological difficulties in adulthood. Such adverse experiences in the developmental years, sometimes for prolonged periods, could have an impact on their emotional, social and psychological resources. This impact is heightened in CSA adult survivors as the interpersonal nature of harm could reverberate throughout their adult relationships, with complex emotional responses to traumatic stressors. Despite the demonstrated effectiveness of trauma focused treatments in the West, culturally specific understanding of the needs and treatments for such survivors in South Asia is still in its infancy. This is important to address their meaning of presenting complaints in South Asia and offering them treatments suitable for them.

**Objectives:**

In this systematic review, we aimed to synthesize the findings of existing research on the impact of CSA on adult survivors in South Asia and the current approaches used to treat them.

**Methods:**

We searched nine databases and ‘hand searched’ important peer-reviewed journals published in South Asian countries from inception until 3rd April 2022. Searches focused on adult survivors of CSA of South Asian origin residing in South Asia, different treatments offered and the efficacy and acceptability of these treatments.

**Results:**

We identified and screened 2608 records and included 56 articles in our full text screening. Out of those, we included 22 articles in the final review. Studies were from four out of the eight countries in South Asia; India, Sri Lanka, Nepal and Pakistan. Of note, only six of those studies focused exclusively on CSA whereas others included all forms of abuse and neglect. All except one article explored experiences of survivors. Only one article focused on their recovery process, within and/or outside of professional treatment with no published research on treatments for them in South Asia. Physical abuse and emotional abuse or neglect were more often reported in our included studies as compared to sexual abuse.

**Conclusions:**

Our review suggests that even though the needs of adult CSA survivors in South Asia have been partly identified, there is very little research into the treatment of CSA adult survivors in this region. Perpetrators often come from their immediate or extended families, leading to their distrust of the familial and legal systems. Even when not diagnosed with a severe mental health condition, there are potentially serious implications for victims’ adult relationships and social functioning. There is a current lack of research and therefore, lack of evidence-based treatment for adult survivors of CSA in South Asia.

**Disclosure of Interest:**

None Declared

